# Comparison of GnRH agonist *versus* luteal estradiol GnRH antagonist protocol using transdermal testosterone in poor responders

**DOI:** 10.5935/1518-0557.20180090

**Published:** 2019

**Authors:** Francesc Fàbregues, Roser Solernou, Janisse Ferreri, Marta Guimerá, Sara Peralta, Gemma Casals, Joana Peñarrubia, Montserrat Creus, Dolors Manau

**Affiliations:** 1 Institut Clinic de Ginecologia, Obstetricia y Neonatología (ICGON). Hospital Clinic de Barcelona. Institut de Investigacions Biomédiques August Pi iSunyer (IDIBAPS)

**Keywords:** estradiol priming, poor responder, Bologna criteria, transdermal testosterone, GnRH analogues, ovarian stimulation

## Abstract

**Objective:**

Transdermal testosterone has been used in different doses and in different stimulation protocols in poor responders. The aim of the present study is to compare the luteal estradiol/GnRH antagonists protocol *versus* long GnRH agonists in poor responder patients according to the Bologna criteria, in which transdermal testosterone has been used prior to the stimulation with gonadotropins.

**Methods:**

In this retrospective analysis, a total of 141 poor responder patients according to the Bologna criteria were recruited. All patients were treated with transdermal testosterone preceding ovarian stimulation with gonadotropins during 5 days. In 53 patients we used the conventional antagonist protocol (Group 1). In 88 patients (GrH pituitary suppression was achieved by leuprolide acetate according to the conventional long protocol (Group 2). We analyzed the ovarian stimulation parameters and IVF outcomes.

**Results:**

Comparing groups 1 and 2, there were no significant differences between cancellation rates and number of oocytes retrieved. However the total gonadotropin dose used and the mean length of stimulation were significantly lower in group 1 when compared to group 2. There were no significant differences in pregnancy outcomes; however, there was a slight increase in the implantation rate in group 1 vis-a-vis group 2, although statistical significance was not achieved.

**Conclusion:**

TT in poor responder patients can be effective both with the conventional agonist's long protocol and with the conventional antagonist's protocol. However, short regimes with previous estradiol antagonists in the luteal phase facilitate ovarian stimulation by shortening the days of treatment and the consumption of gonadotropins

## INTRODUCTION

Poor response to ovarian stimulation affects a significant proportion of infertile couples seeking fertility advice. Although in the past few years a debate has arisen regarding the definition of poor ovarian response, the European Society of Human Reproduction and Embryology (ESHRE) working group on Poor Ovarian Response Definition recently developed new criteria to define patients who respond poorly to ovarian stimulation; the so called "Bologna criteria" (Ferraretti *et al*., 2011). These criteria incorporate age, ovarian reserve tests (anti-Mullerian hormone-AMH-level or antral follicle count - AFC) and ovarian response in previous IVF/ICSI cycles in the definition, and represent the first realistic attempt by the scientific community (ESHRE) to standardize the definition of poor ovarian response in a simple and reproductive manner.

The first studies published including women with poor ovarian response, according to the Bologna criteria, have shown disappointingly low pregnancy rates, irrespectively of age. A recent observational study demonstrated a very poor prognosis for these women, given that live birth rates following treatment with natural cycle IVF was < 3% per patient, irrespective of age, and significantly lower when compared to women who did not fulfill the Bologna criteria (Polyzos *et al*., 2012).

A poor response to ovulation stimulation results in high cancellation rates of up to 76% and extremely low pregnancy rates, from 3.2-14% (Ulug *et al*., 2003; Busnelli *et al*., 2015). Various strategies for poor responders, including agonist and antagonist protocols have been attempted; however, at present, there is no definitive evidence that poor outcomes can be reversed by a specific protocol (Ubaldi *et al*., 2014; Ata & Seli, 2015).

It has been suggested that the buildup of androgens in the micro milieu of the primate ovary, plays a critical role in early follicular development and granulosa cell proliferation, and increase the number of preantral and antral follicles (Weil *et al*., 1999; Hillier *et al*., 1997). In addition, increased intraovarian concentration of androgens seems to augment follicle stimulating hormone (FSH) receptor expression in the granulosa cells (Vendola *et al*., 1998; 1999).

Based on the limited available evidence, transdermal testosterone pretreatment seems to increase clinical pregnancy and live birth rates in poor responders undergoing ovarian stimulation for IVF (Ata & Seli, 2015; González-Comadran *et al*., 2012). However, there is insufficient data to support a beneficial role of rLH, hCG, DHEA or letrozole administration in the probability of pregnancy in poor responders undergoing ovarian stimulation for IVF (Bosdou *et al*., 2012).

Transdermal testosterone (TT) has been used at different doses and in different stimulation protocols (Bosdou *et al*., 2016; Kim *et al*., 2011; Fàbregues *et al*., 2009; Massin *et al*., 2006). However, it is difficult to establish its efficacy with sufficient evidence (Polyzos *et al*., 2018). This study compared luteal estradiol/GnRH antagonists protocol *versus* long GnRH agonists in poor responder patients according to the Bologna criteria, in which transdermal testosterone has been used prior to the stimulation with gonadotropins.

## MATERIALS AND METHODS

### Patients

This study was performed by a retrospective analysis of our database of women referred to our center for IVF, and was conducted from January 2015 to May 2016 in the Assisted Reproduction Unit of the Hospital Clinic in Barcelona (Spain). We recruited 141 poor responder patients according to the Bologna criteria.

All the patients were in good health within normal limits of thyroid, kidney and hepatic laboratory results, and they had regular menstruation periods with duration of 21-35 days. None of them had taken any infertility medication in the 3 months prior to the study.

**Figure 1:**
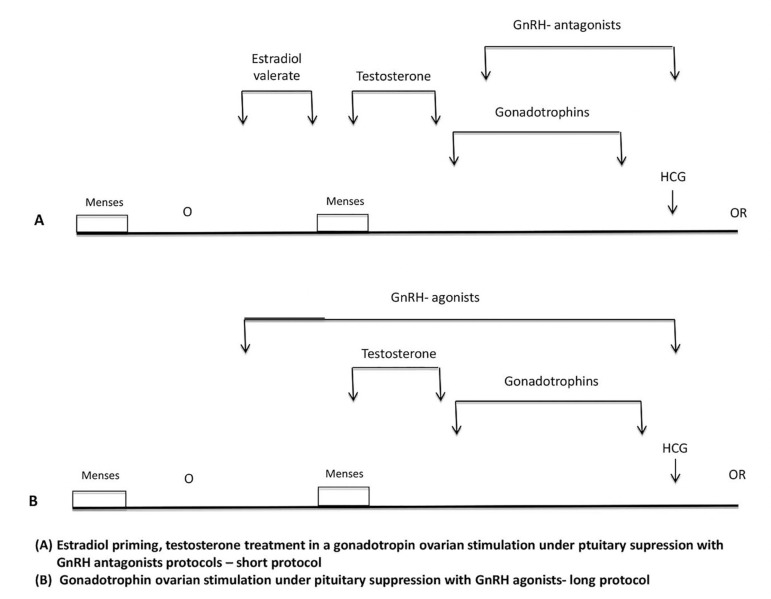
Schematic representation protocols.

The use of agonists or antagonists depended on the criterion of the specialist that indicated the treatment; however, the pattern of androgenization was similar in both groups of patients. All patients were treated with transdermal testosterone (TT) preceding ovarian stimulation with gonadotropins, but in one group we used luteal estradiol valerate and the GnRH antagonist protocol (Group 1); whereas in the second group (Group 2) we used the long GnRH agonist protocol (Fig. 1). The study was approved by our Institutional Review Board and informed consent was obtained from all individual participants included in the study (HB-15-EL-RS-C).

Study parameters, including days of stimulation, dose of gonadotropin administered, peak E2 level on the day of human chorionic gonadotropin (hCG) administration, number of oocytes retrieved , number of embryos and high quality embryos were evaluated. Pregnancy outcomes, including clinical and ongoing pregnancy rates were also analyzed.

In no cycle we performed preimplantational diagnosis

### Stimulation regimens 

All patients included in the study performed the same pattern with transdermal testosterone (TT). Testosterone therapy was commenced on the first day of the next menstrual cycle in Group 1, whereas in Group 2 testosterone began on the day when pituitary-ovarian suppression was confirmed. The therapy with testosterone was continued for 5 days.

Transdermal testosterone treatment was carried out using a daily single patch with a 2.5 mg/day nominal delivery rate of testosterone (Testopatch, Pierre Fabre Iberica SA, Barcelona, Spain) which was applied on the thigh at night and removed always at 09:00h in the morning.

This transdermal delivery system maintains stable testosterone levels within narrow ranges with little within - and between - subject variation, providing a highly controllable way of delivering testosterone reliably, and the hormonal dose administered can be modified according to the duration of patch application (Buckler *et al*., 1998; De Sanctis *et al*., 1998; Mazer, 2000). We chose to use testosterone 20 mg/kg per day for 5 days on the basis of previous experimental studies in primates (Vendola *et al*., 1998; 1999).

Thus, in each patient, the patch was applied at night at a time aimed to leave it in place for a predetermined number of hours in order to provide the desired daily dose of testosterone (e.g. in a woman weighing 60kg and needing 1200mg/day, the patch was used for 12h [0.1mg/h delivery rate 12h. 1.2mg or 1200mg] and thus applied at 21:00h). Testosterone therapy was performed according to a routinely used protocol (Balasch *et al*., 2006; Fàbregues *et al*., 2009).

In 53 patients (Group 1), estradiol priming (4mg of oral estradiol valerate (E_2_) (Progynova; Bayer, Spain)) was initiated on luteal day 21th and stopped in the first day of the next menstrual cycle. After TT therapy, recombinant FSH (Gonal-F, Merck S.A., Madrid, Spain.) was initiated at an initial dose of 300IU/day together with 75IU HMG (Menopur, Ferring SA, Madrid, Spain). The gonadotropin dose was adjusted according to serum E2 levels and serial ultrasound monitoring. The GnRH antagonist (Cetrotide, Merck S.A., Madrid, Spain) was administered at a dose of 250µg/0.5ml/day when the leading follicle reached 14-15mm in its maximum diameter. GnRH administration continued until the day of hCG injection.

In 88 patients (Group 2), pituitary suppression was achieved by subcutaneous administration of leuprolide acetate (Procrin; Abbott Laboratories, Madrid, Spain). This treatment was started in the mid-luteal phase of the previous cycle and given 1 mg daily, then reduced to 0.5mg after ovarian arrest, when serum estradiol (E2) concentration declined to < 50pg/ml and a vaginal ultrasound scan showed an absence of 10mm-diameter follicles. Transdermal testosterone was administered during 5 days and gonadotropin ovarian stimulation was started the day following the last testosterone patch application. On Days 1 to 4 of ovarian stimulation, 300IU per day of r-hFSH (Gonal-F, Merck S.A., Madrid, Spain) together with 75IU HMG (Menopur, Ferring S.A., Madrid, Spain) were administered. On day 5 onward, the gonadotropin dose was administered on an individual basis according to ovarian response.

The criteria for hCG administration (250mg s.c.Ovitrelle, Serono S.A.) were the presence of two or more follicles >18 mm in diameter, with >4 follicles measuring >14 mm in association with a consistent rise in serum E2 concentration. The cycle was cancelled when there were less than 3 follicles with diameter >14 mm after 8-9 days of gonadotropin therapy, or after 4-5 additional treatment days without attaining, or the imminent prospect of attaining, the criteria for hCG administration.

Oocyte aspiration was performed with vaginal ultrassonography 35-36h after hCG administration. Embryo grading was recorded according to published criteria (Veeck, 1999); embryos graded 1 or 2 were considered of high quality. In both groups, embryo transfer was performed in the cleavage stage (day 3). The luteal phase was supported with vaginal micronized progesterone (600mg/day given at 8h intervals) starting on the day following oocyte aspiration and continuing either up to menstruation or, if the patients became pregnant, for at least the first 3 weeks of pregnancy.

Pregnancy was diagnosed by a positive serum β-hCG test 12 days after ET. Clinical pregnancy was defined by observation of a fetal heartbeat using transvaginal ultrasonography at 5-6 weeks gestation.

### Statistical analysis

All statistical analyses were performed using the SPSS version 23.0 software (Chicago, IL, USA). We used a t-test to compare the mean values between two different stimulation protocols.

Differences in outcome rates were analyzed using an χ^2^ test or Fisher's exact test. *p*<0.05 was considered statistically significant.

## RESULTS

Table 1 depicts the baseline characteristics of the patients enrolled in the two different stimulation protocols. The groups were similar with respect to age, body mass index (BMI), duration of infertility, antral follicle count, AMH levels and basal FSH and estradiol.

**Table 1:** Comparison of patient characteristics for cycles using Luteal E_2_/TT/GnRH antagonist vs. TT/GnRH agonist protocol

Variable	Group 1	Group 2	*p*
	(Luteal E_2_/TT/GnRH antagonist)	(TT/GnRH agonist)	
	(n=53)	(n=88)	
Age (years)	37.06±0.4	36.09±0.2	NS
BMI (Kg/m^2^)	24.25±0.7	24.33±0.6	NS
Duration of infertility (years)	5.0±1.3	4.9±1.6	NS
Cause of infertility			
Male factor (n ;%)	16 (30.1)	33 (37.5)	NS
Unexplained (n ;%)	17 (32.2)	25 (28.4)	NS
Endometriosis (n ;%)	13 (24.5)	20 (22.8)	NS
Tubal factor (n ;%)	7 (13.3)	10 (11.3)	NS
Baseline FSH (UI/L)	11.1±0.7	11.5±0.4	NS
Baseline Estradiol (pg/ml)	55.04±4.8	50.02±2.5	NS
AMH (ng/ml)	0.8±0.1	1.0±0.2	NS
Antral follicle count (n)	5.3±0.5	5.6±0.3	NS
Previous cycles with poor response (n)*	10	18	NS

Values are mean ± DE unless specified otherwise

* Including cancelled cycles and cycles with ≤3 oocytes collected

There were no reported major side effects after testosterone therapy and two protocols were well-tolerated by all patients.

Table 2 shows the stimulation parameters in both groups studied. The number of cancelled cycles due to inadequate response was similar 13.6% *vs.* 15.1%. The number of follicles and estradiol levels on hCG day were not significantly different. However, the total gonadotropin dose used was significantly higher (2709±123IU *vs.* 2258±13; *p*=0.023) in group 2 compared to group 1. In addition the mean length of stimulation was significantly higher (9.5±0.2 *vs.* 7.9±0.3 days; *p*=0.001) in group 2, when compared to group 1.

**Table 2:** Ovarian stimulation characteristics in Groups 1 and 2

Variable	Group 1	Group 2	*p*
	(Luteal E_2_/TT/GnRH antagonist)	(TT/GnRH agonist)	
	(n=53)	(n=88)	
Days of stimulation	7.95±0.36	9.59±0.26	0.001
Total UI of FSH	2258±136	2709 ±123	0.023
Patients with HCG and ovum retrieval (n,%)	45 (84.9%)	76 (86.4%)	0.805
No. of follicle in hCG day			
- 10-14 mm	1.17±0.18	1.44±0.16	0.275
- >14-<18 mm	1.65±0.20	2.03±0.21	0.222
- ≥18mm	2.42±0.20	2.77±0.20	0.254
Estradiol on hCG day (pg/ml)	1235±102.4	1495±99.0	0.082

Values are mean ± DE unless specified otherwise

When comparing ovum retrieval and IVF outcomes in groups 1 and 2, there were no significant differences. However, there was a trend towards a slight improvement in the implantation rate 27.3% *vs.* 19%, pregnancy rate per oocyte retrieval (37.8% *vs.* 31.6%) and per embryo transfer (38.6% *vs.* 34.3%) in group 1 as compared with group 2 (Table 3).

**Table 3:** Ovum retrieval and IVF/ICSI outcome in groups 1 and 2

Variable	Group 1	Group 2	*p*
	(Luteal E_2_/TT/GnRH antagonist)	(TT/GnRH agonist)	
	(n=53)	(n=88)	
Patients with hCG and ovum retrieval (n ;%)	45 (84.9)	76 (86.4)	0.80
No. oocytes	4.41±0.39	4.83±0.29	0.390
No. of metaphase II oocytes	3.11±0.34	3.83±0.27	0.104
No. of 2pn oocytes on day 1	3.11±0.27	2.91±0.26	0.602
No. of patients with embryo transfer (n, %)	42 (83.0%)	70 (79.5%)	0.610
No. of embryos per replacement	1.75±0.09	1.73 (±0.70)	0.854
High quality embryos replaced	1.1±0.1	1.1±0.2	0.625
Implantation rate (%)	27.3	19	0.187
Clinical pregnancies			
-Number	17	24	-
-Per started cycle (%)	32.1	27.3	0.742
-Per oocyte retrieval (%)	37.8	31.6	0.683
-Per embryo transfer (%)	38.6	34.3	0.780
-Multiple pregnancies (n, %)	6 (13.6)	3 (3.4)	0.231
-Miscarriages (n, %)	3 (5.6)	3 (3.4)	0.735
-OHSS (n, %)	-	-	-

Values are mean ± unless specified otherwise

## DISCUSSION

This is the first study comparing different GnRH analogues protocols in poor responder patients according to the Bologna criteria in which TT has been used. The potential stimulating role of androgens on folliculogenesis has been suggested by a number of basic research studies (Weil *et al*., 1999; Vendola *et al*., 1999; Hillier *et al*., 1997), and illustrated by some pathophysiological conditions (Norman, 2002; Pigny *et al*., 2003) and clinical models (Nagels *et al*., 2015; Grynberg *et al*., 2010; Futterweit & Deligdisch, 1986). Transdermal testosterone has been shown in previous small RCTs to increase the reproductive outcomes of IVF/ICSI patients (González-Comadran *et al*., 2012). In most of these studies, transdermal testosterone in relatively high doses was administered before ovarian stimulation with a duration varying from 5 to 21 days (Bosdou *et al*., 2016; Kim *et al*., 2011; Fàbregues *et al*., 2009; Massin *et al*., 2006).

Several previous studies have shown that testosterone may indeed have a role during the later stages of follicular growth by increasing follicle-stimulating hormone receptor messenger RNA in preovulatory follicles, and by stimulating oocyte maturation. However, most of the published experiments indicate that testosterone mainly acts during the earlier stages of folliculogenesis by playing a role in follicle activation and growth (Walters, 2015).

In this study we chose to use TT for 5 days on the basis of studies in primates and also available reports from previous clinical studies (Fàbregues *et al*., 2009; 2013; Balasch *et al*., 2006). Studies suggest that IGF-I appears to mediate or facilitate the effect of TT on early follicle development, and also improves oocyte and embryo quality (Meldrum *et al*., 2013). IGF-I stimulation by testosterone may explain the unusually high implantation rates reported in some studies with treatments aimed at increasing the exposure of any kind of testosterone to ovarian follicles in poor responders (Bosdou *et al*., 2012; Kim *et al*., 2011).

Regardless of the dose and duration of the treatment with TT, it has been used both in long GnRH agonist (Bosdou *et al*., 2016; Walters, 2015; Fàbregues *et al*., 2009) and short GnRH antagonist protocols (Doan *et al*., 2017; Kim *et al*., 2011; Massin *et al*., 2006), but these protocols have never been compared in this context before. Several studies suggested that there was no significant difference on the number of oocytes retrieved, mature oocytes and pregnancy rates in both GnRH antagonist and GnRH agonist protocols in poor responders (Pandian *et al.,* 2010; Devesa *et al.,* 2010). However, Pu *et al*. (2011) demonstrated that the stimulation duration was significantly lower with the GnRH antagonist protocol. The results of our study coincide with that provided in the literature in the sense that the use of TT in a GnRH antagonist protocol could be a useful option in these patients, shortening the duration of stimulation and the quantity of gonadotropins used.

Several studies suggested that luteal estradiol could improve the results in poor responders, shortening GnRH antagonist stimulation cycles (Chang *et al.,* 2012)*,* decreasing cancellation cycles (Reynolds *et al*., 2013), and improving FSH effects in granulosa cells (Ireland & Richards, 1978; Wang & Greenwald, 1993). In our study it has not been possible to evaluate the luteal estradiol efficacy, because we did not have a control group in which we used the antagonist protocol without previous estradiol. However, taking into account what is suggested in the literature, this could be a valid treatment option that should be analyzed in subsequent randomized studies.

The main limitation of this study was its retrospective design and small sample size. However, the poor responder population according to the Bologna criteria represents only a 5 to 10% of patients in most assisted reproduction clinics, which creates logistic problems when performing a prospective study with sufficient power. Although the patients were not randomized, the two populations had similar baseline characteristics, which made possible to compare IVF outcomes between the groups.

Adjuvant therapy with TT can be used with similar efficacy with both GnRH agonist and GnRH antagonist protocols in poor responders. More studies are needed to analyze whether luteal estradiol can improve the response profile when TT is applied in GnRH antagonist protocol in these patients.

## CONCLUSIONS

Although there are controversial aspects regarding androgenic therapy in low-responders, it seems that it can be a valid option as adjuvant therapy to gonadotropins. Its efficacy is not significantly different when different GnRH analogues are used; however, short regimes with antagonists with previous estradiol in the luteal phase facilitate ovarian stimulation by shortening the days of treatment and gonadotropin use.
